# GUCY2C maintains intestinal LGR5^+^ stem cells by opposing ER stress

**DOI:** 10.18632/oncotarget.22084

**Published:** 2017-10-26

**Authors:** Crystal L. Kraft, Jeffrey A. Rappaport, Adam E. Snook, Amanda M. Pattison, John P. Lynch, Scott A. Waldman

**Affiliations:** ^1^ Department of Pharmacology and Experimental Therapeutics, Thomas Jefferson University, Philadelphia, United States of America, PA, USA; ^2^ Division of Gastroenterology, Department of Medicine, Abramson Cancer Center, University of Pennsylvania, Philadelphia, United States of America, PA, USA

**Keywords:** intestinal stem cell, guanylate cyclase C, Lgr5, Bmi1, epithelial regeneration

## Abstract

Long-lived multipotent stem cells (ISCs) at the base of intestinal crypts adjust their phenotypes to accommodate normal maintenance and post-injury regeneration of the epithelium. Their long life, lineage plasticity, and proliferative potential underlie the necessity for tight homeostatic regulation of the ISC compartment. In that context, the guanylate cyclase C (GUCY2C) receptor and its paracrine ligands regulate intestinal epithelial homeostasis, including proliferation, lineage commitment, and DNA damage repair. However, a role for this axis in maintaining ISCs remains unknown. Transgenic mice enabling analysis of ISCs (*Lgr5-GFP*) in the context of GUCY2C elimination (*Gucy2c*^*–/–*^) were combined with immunodetection techniques and pharmacological treatments to define the role of the GUCY2C signaling axis in supporting ISCs. ISCs were reduced in *Gucy2c*^*–/–*^ mice, associated with loss of active Lgr5^+^ cells but a reciprocal increase in reserve Bmi1^+^ cells. GUCY2C was expressed in crypt base Lgr5^+^ cells in which it mediates canonical cyclic (c) GMP-dependent signaling. Endoplasmic reticulum (ER) stress, typically absent from ISCs, was elevated throughout the crypt base in *Gucy2c*^*–/–*^ mice. The chemical chaperone tauroursodeoxycholic acid resolved this ER stress and restored the balance of ISCs, an effect mimicked by the GUCY2C effector 8Br-cGMP. Reduced ISCs in *Gucy2c*^*–/–*^mice was associated with greater epithelial injury and impaired regeneration following sub-lethal doses of irradiation. These observations suggest that GUCY2C provides homeostatic signals that modulate ER stress and cell vulnerability as part of the machinery contributing to the integrity of ISCs.

## INTRODUCTION

The intestinal epithelium is highly dynamic, undergoing continuous cycles of renewal and repair. Stem cells at the base of crypts give rise to progenitor cells that continue to divide, migrate up the crypt-villus axis, and differentiate into the specialized epithelial cell types of the intestine [1]. Absorptive cells are sloughed off into the intestinal lumen in a conveyor belt fashion on a weekly basis, while secretory cells such as tuft cells and Paneth cells survive for weeks [2, 3]. Beyond this programmed turnover, intestinal insults, such as inflammation, oxidative damage, and radiation [4, 5] induce cell death, requiring replacement to maintain the epithelial barrier. These processes of turnover and regeneration are driven by an equally dynamic population of intestinal stem cells (ISCs) whose characteristics are only beginning to emerge [2].

The highly organized ISC compartment at the base of crypts contains cell types with distinct marker expression and functional phenotypes. Lgr5^+^, or crypt base columnar (CBC), cells are long-lived multipotent stem cells located at crypt cell positions 0–4 that divide daily to drive weekly turnover of the epithelium, making them the “active” stem cells [6]. These cells are exquisitely sensitive to insult and are intimately associated with differentiated cells that supply essential regulatory signals, including Paneth cells [6-8]. Another long-lived, multipotent stem cell type located higher up the crypt axis around cell positions 4–8 commonly expresses the marker Bmi1 [9]. These Bmi1^+^ cells are quiescent and contribute minimally to tissue homeostasis [6]. However, upon injury, Bmi1^+^ cells can restore both the more active CBCs as well as all of the differentiated cell types of the intestinal epithelium, earning them the label of “reserve” ISC [6, 10]. Despite the sensitivity of Lgr5^+^ cells to death upon intestinal insult and the contribution of Bmi1^+^ cells to regeneration, Lgr5^+^ cells are required for recovery from radiation-induced gastrointestinal damage [5]. While the identity and function of intestinal stem cell populations are emerging, mechanisms contributing to their maintenance and relative balance continue to be refined [6-8, 11].

GUCY2C is a membrane-associated guanylate cyclase receptor selectively expressed in apical membranes of intestinal epithelial cells from the duodenum to the distal rectum [12]. Cognate ligands are structurally similar peptides and include the paracrine hormones guanylin, produced throughout the intestine, and uroguanylin, produced selectively in small intestine, and the heat-stable enterotoxins (STs) produced by diarrheagenic bacteria [12]. GUCY2C originally was identified as a mediator of intestinal fluid and electrolyte secretion contributing to the pathophysiology of enterotoxigenic diarrhea [12]. However, the GUCY2C-paracrine hormone axis has emerged as an essential regulator of key homeostatic processes, including cell proliferation [13, 14], lineage commitment [15], and DNA damage repair [14], functions that are essential to the integrity of the crypt [16]. Further, in murine models of tumorigenesis or inflammatory bowel disease, in which injury and recovery characteristically involve ISCs [17], silencing GUCY2C amplifies pathophysiology, tissue damage, and mortality [14, 18–21]. Here, we explore the role for GUCY2C signaling in maintaining ISCs.

## RESULTS

### Eliminating GUCY2C expression disrupts ISC numbers

**Figure 1:**
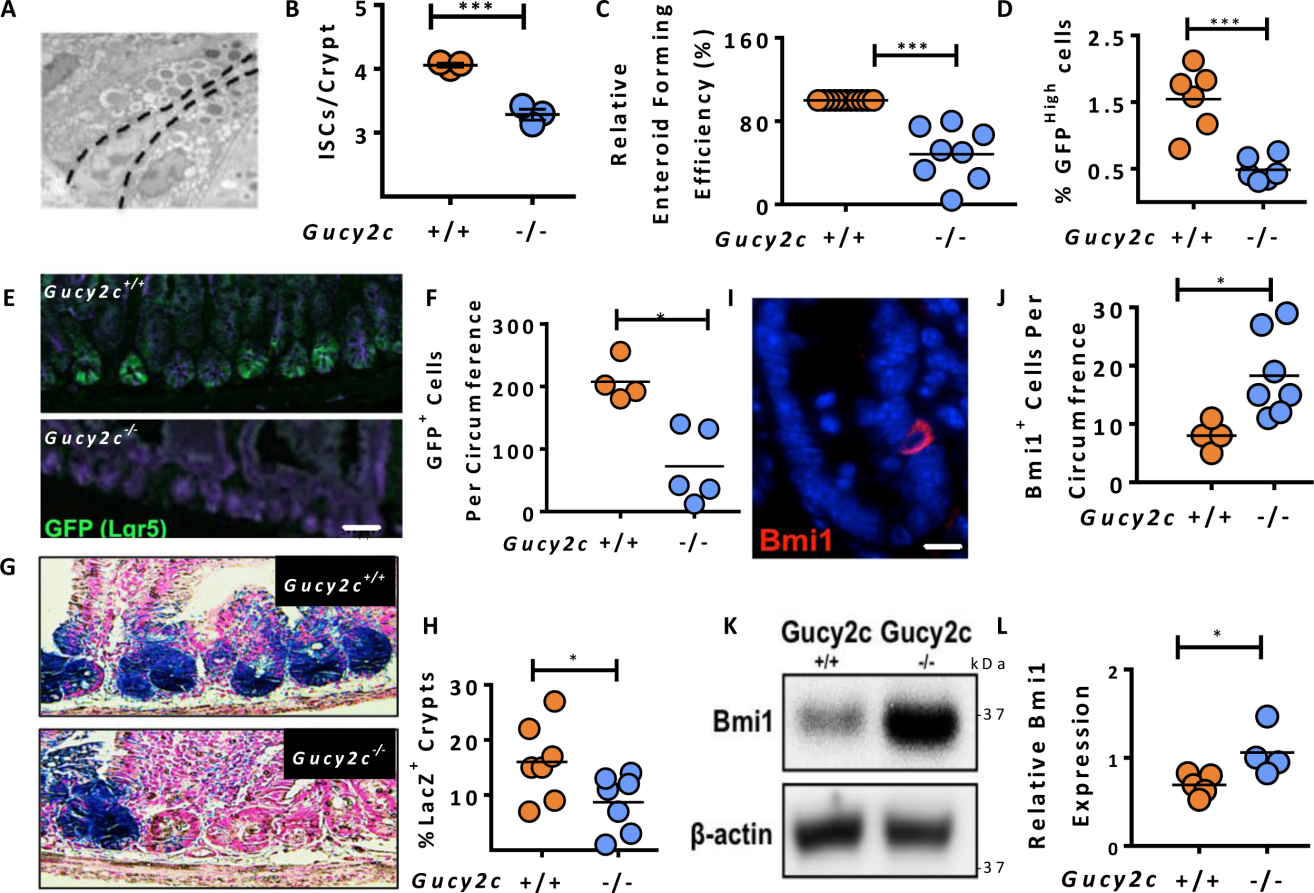
Gucy2c maintains the balance of Lgr5^+^ and Bmi1^+^ cells in crypts (**A**–**B**) Enumeration of CBC ISCs in small intestinal sections using transmission electron microscopy (*n* = 3 mice, >30 crypts/mouse). (**C**) *Ex vivo* enteroid forming capacity of crypts from *Gucy2c*^***–/–***^ mice relative to *Gucy2c*^***+/+***^ mice. (**D**) Quantification of Lgr5^+^ (GFP^High^) cells by flow cytometry in crypts from *Lgr5-EGFP-Cre-Gucy2c*^***+/+***^ and *Gucy2c*^***–/–***^ mice. (**E**–**F**) Enumeration of Lgr5^+^GFP^+^ cells in intestinal crypts by EGFP IF (>4 sections/mouse). (**G**–**H**) Crypt Lgr5^+^ cell lineage tracing events expressed as a percent of total crypts per section (>4 sections/mouse). (**I**–**J**) Bmi1^+^ cells per intestinal section (>4 sections/mouse). (**K**–**L**) Quantification of Bmi1 expressed in isolated crypt lysates, relative to β-actin (*n* = 5 *Gucy2c*^***+/+***^, 4 *Gucy2c*^***–/–***^). *p < 0.05; ***p < 0.001. Bars in E and G represent 50 µm; bar in I represents 20 µm.

Stem cells were enumerated in small intestinal crypts from *Gucy2c*^***+/+***^ and *Gucy2c*^***–/–***^ mice by electron microscopy. Wedge-shaped cells in crypt positions 0 to 5 were included, and Paneth cells were excluded by their vesicular morphology (Figure 1A) [6–8]. The total number of ISCs in the crypt base was reduced in the absence of GUCY2C (Figure 1B). Similarly, *ex vivo* enteroid formation, a measure of ISC number and function [22], was reduced in the absence of GUCY2C ( *p* < 0.001; Figure 1C). FACS analyses revealed fewer Lgr5^+^/GFP^High^ cells in *Lgr5-EGFP-IRES-CreERT2* mice in which GUCY2C was eliminated (*Lgr5-EGFP-Cre-Gucy2c*^***–/–***^; Supplementary Figure 1 and Figure 1D), confirmed by immunofluorescence microscopy (Figure 1E–1F and Supplementary Figure 2). Moreover, lineage tracing in *Lgr5-EGFP-Cre-Gucy2c*^***+/+***^ and –*Gucy2c*^***–/–***^ mice crossed onto the *Rosa-STOP*^***fl***^*-LacZ* background revealed that *Gucy2c*^***–/–***^ mice had fewer LacZ-labeled crypts (Figure 1G–1H). Conversely, *Gucy2c*^***–/–***^ mice exhibited an expanded population of Bmi1^+^ cells by immunofluorescence microscopy (Figure 1I–1J and Supplementary Figure 3) which was confirmed by immunoblot analysis (Figure 1K–1L). Together, these results suggest that eliminating GUCY2C signaling rebalances stem cell populations, favoring a “reserve” ISC phenotype.

### Functional expression of the GUCY2C signaling axis in ISCs

**Figure 2:**
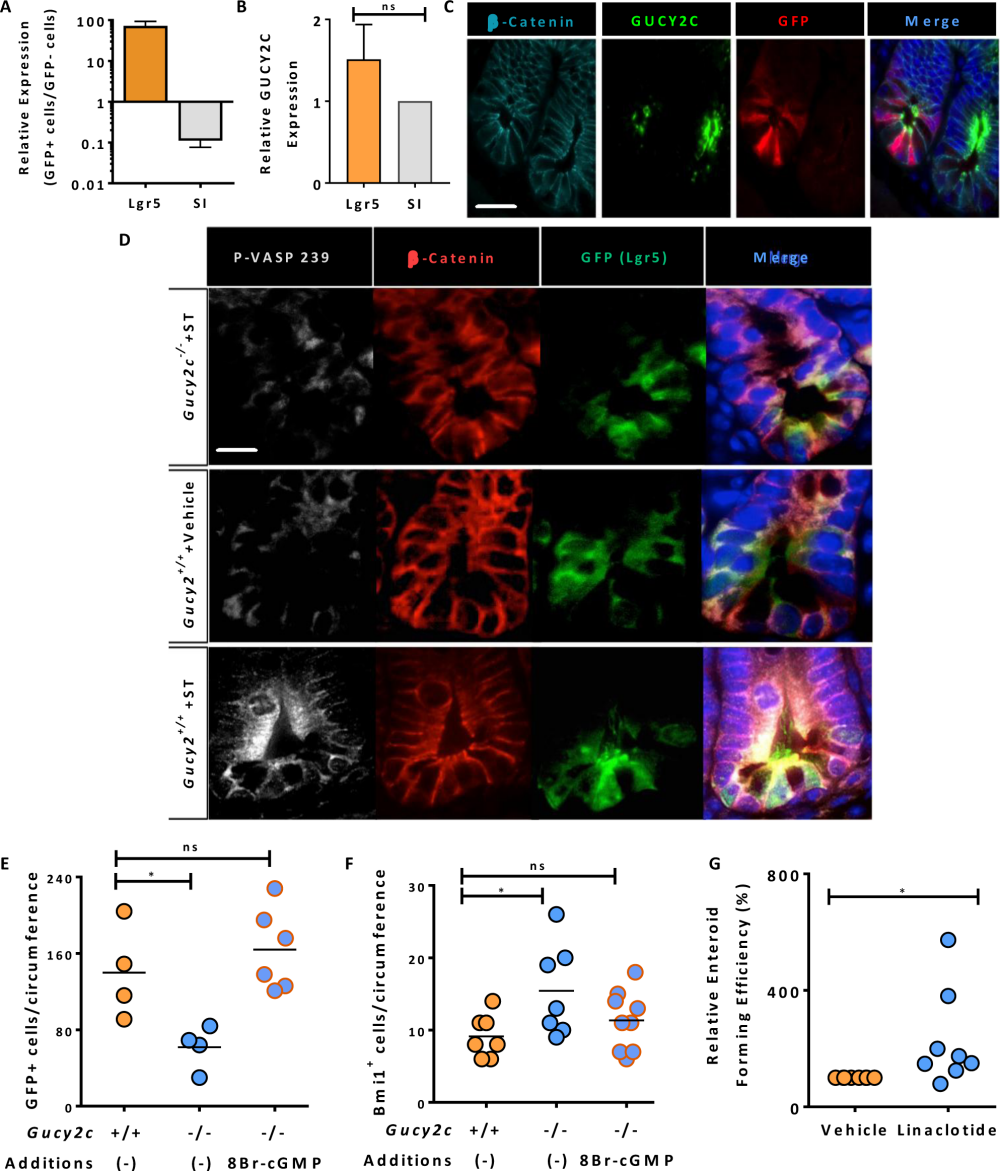
Functional GUCY2C is expressed in Lgr5^+^ cells (**A**) Flow sorting of GFP^+^ and GFP^–^ cells from crypts of *Lgr5-EGFP-Cre-Gucy2c*^***+/+***^ mice produced populations of active stem (Lgr5^High^/SI^Low^) and differentiated (Lgr5^Low^/SI^High^) cells (*n* = 3). (**B**) GUCY2C mRNA expression, quantified by RT-PCR, was compared in Lgr5^High^/SI^Low^ and Lgr5^Low^/SI^High^ cells. (**C**) GUCY2C (green), immunofluorescence in GFP^+^ (red) cells. β-catenin (cyan) highlights individual cells and DAPI (blue) highlights nuclei. (**D**) ST activates GUCY2C and downstream VASP serine 239 phosphorylation (P-VASP-239) (white) in GFP^+^ (green) cells in *Gucy2c*^***+/+***^, but not *Gucy2c*^***–/–***^, mice. β-catenin (red) highlights individual cells and DAPI (blue) highlights nuclei. (**E**–**F**) 8Br-cGMP reconstitutes levels of (E) Lgr5^+^GFP^+^ and (F) Bmi1^+^ cells in crypts of *Gucy2c*^***–/–***^ mice that are comparable to those in *Gucy2c*^***+/+***^ mice. (**G**) Linaclotide enhances the enteroid-forming capacity of crypts in *Gucy2c*^***–/–***^ mice relative to *Gucy2c*^***+/+***^ mice. *p < 0.05; ns, not significant. Bar in C represents 50 µm; bar in D represents 20 µm.

Lgr5^+^GFP^+^ cells were collected by FACS from *Lgr5-EGFP-Cre-Gucy2c*^***+/+***^ and –*Gucy2c*^***–/–***^ mice (Supplementary Figure 4) [23] and enrichment verified by RT-qPCR of stem (Lgr5) and differentiated cell [sucrose isomaltase (SI)] mRNA markers (Figure 2A). Expression of *Gucy2c* mRNA in stem (Lgr5^High^/SI^Low^) cells was quantitatively similar to that of differentiated (Lgr5^Low^/SI^High^) cells suggesting similar levels of expression in stem cell and differentiated compartments (Figure 2B). Immunofluorescence microscopy confirmed specific co-localization of GUCY2C in Lgr5^+^GFP^+^ stem cells (Figure 2C and Supplementary Figure 5). To confirm functionality of the GUCY2C receptor in ISCs, ST was injected into segments of intestinal lumen of *Lgr5-EGFP-Cre-Gucy2c*^***+/+***^ and *Lgr5-EGFP-Cre*-*Gucy2c*^***–/–***^ mice [24]. Luminal exposure to this GUCY2C agonist [25] produced cGMP accumulation and cGMP-specific phosphorylation of the downstream target of cGMP-dependent protein kinase, vasodilator-stimulated phosphoprotein (VASP), in Lgr5^+^GFP^+^ cells, in *Gucy2c*^***+/+***^, but not in *Gucy2c*^***–/–***^, mice (Figure 2D) highlighting the functionality of GUCY2C in ISCs. Further, 8Br-cGMP, a cell-permeable analog of the GUCY2C second messenger cGMP [26], restored the balance of ISCs, returning Lgr5^+^GFP^+^ (Figure 2E) and Bmi1^+^ (Figure 2F) cells in *Gucy2c*^***–/–***^ mice to levels that were comparable to those in *Gucy2c*^***+/+***^ mice. Moreover, the oral GUCY2C agonist linaclotide (*Linzess™*, Ironwood, Cambridge, MA) amplified the efficiency of enteroid formation in *Gucy2c*^***+/+***^ mice (Figure 2G). These observations reinforce the role of GUCY2C signaling in maintaining ISCs.

### GUCY2C signaling opposes crypt ER stress

**Figure 3:**
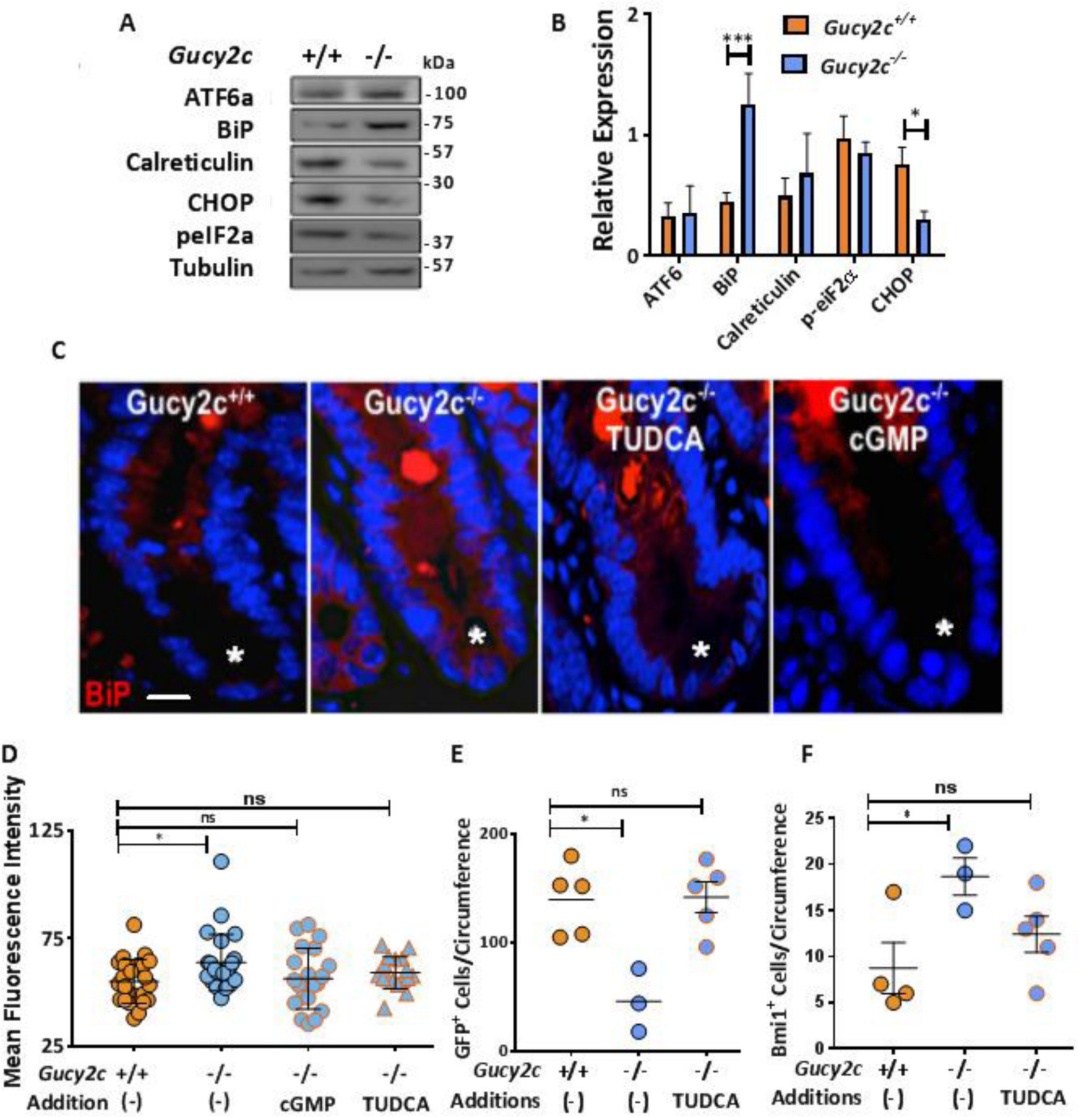
GUCY2C opposes ER stress balancing active and reserve ISCs (**A**, **B**) Quantification of crypt ER stress marker expression, relative to tubulin, in *Gucy2c*^***+/+***^ and *Gucy2c*^***–/–***^ mice (*n* = 3). (**C**, **D**) Grp78 (BiP) expression in crypts (*) of *Gucy2c*^***+/+***^ mice, and *Gucy2c*^***–/–***^ mice before and after treatment with TUDCA or 8Br-cGMP. (**E**, **F**) Quantification of crypt Lgr5^+^GFP^+^ and Bmi1^+^ cells in *Gucy2c*^***+/+***^ and *Gucy2c*^***–/–***^ mice following oral TUDCA for 3 d. *p < 0.05; ***p < 0.001; ns, not significant. Bar in C represents 20 µm.

The normal ISC compartment minimizes endoplasmic reticulum (ER) stress, and prolonged exposure induces ISCs to shift from the stem cell compartment into the proliferating progenitor cell pool [27, 28], an effect which is phenocopied by eliminating GUCY2C signaling [13–15, 20, 29]. Here, elimination of GUCY2C expression induced over-expression of the chaperone protein BiP (Grp78), a canonical marker of ER stress [30], in crypts in *Gucy2c*^***–/–***^ mice (Figure 3A–3D). Interestingly, markers of the unfolded protein response induced by ER stress, including ATF6, calreticulin, and phosphorylated eIF2α (p-eIF2α), were unchanged in those crypts [31] (Figure 3A, 3B). Moreover, the pro-apoptotic protein CHOP, which eliminates cells with irreversible ER stress [32], was paradoxically reduced in those crypts (Figure 3A, 3B). This pattern of markers specifically reflects adaptive ER stress, in which chaperones like BiP are over-expressed to relieve chronic ER stress, minimizing the unfolded protein response, while CHOP transcription is down-regulated to prevent cell death [33, 34]. In that context, tauroursodeoxycholic acid (TUDCA), a bile salt that mimics the chaperone protein BiP to reduce ER stress by relieving protein misfolding [35], restored normal BiP expression in crypts in *Gucy2c*^***–/–***^ mice, an effect which was mimicked by 8Br-cGMP (Figure 3C–3D). Moreover, like 8Br-cGMP (Figure 2F–2G), TUDCA also restored Lgr5^+^GFP^+^ (Figure 3E) and Bmi1^+^ (Figure 3F) cells to normal levels in *Gucy2c*^***–/–***^ mice. These observations underscore the role of GUCY2C signaling in opposing ER stress that is essential to maintaining ISCs.

### GUCY2C maintains ISCs supporting regeneration after radiation injury

**Figure 4:**
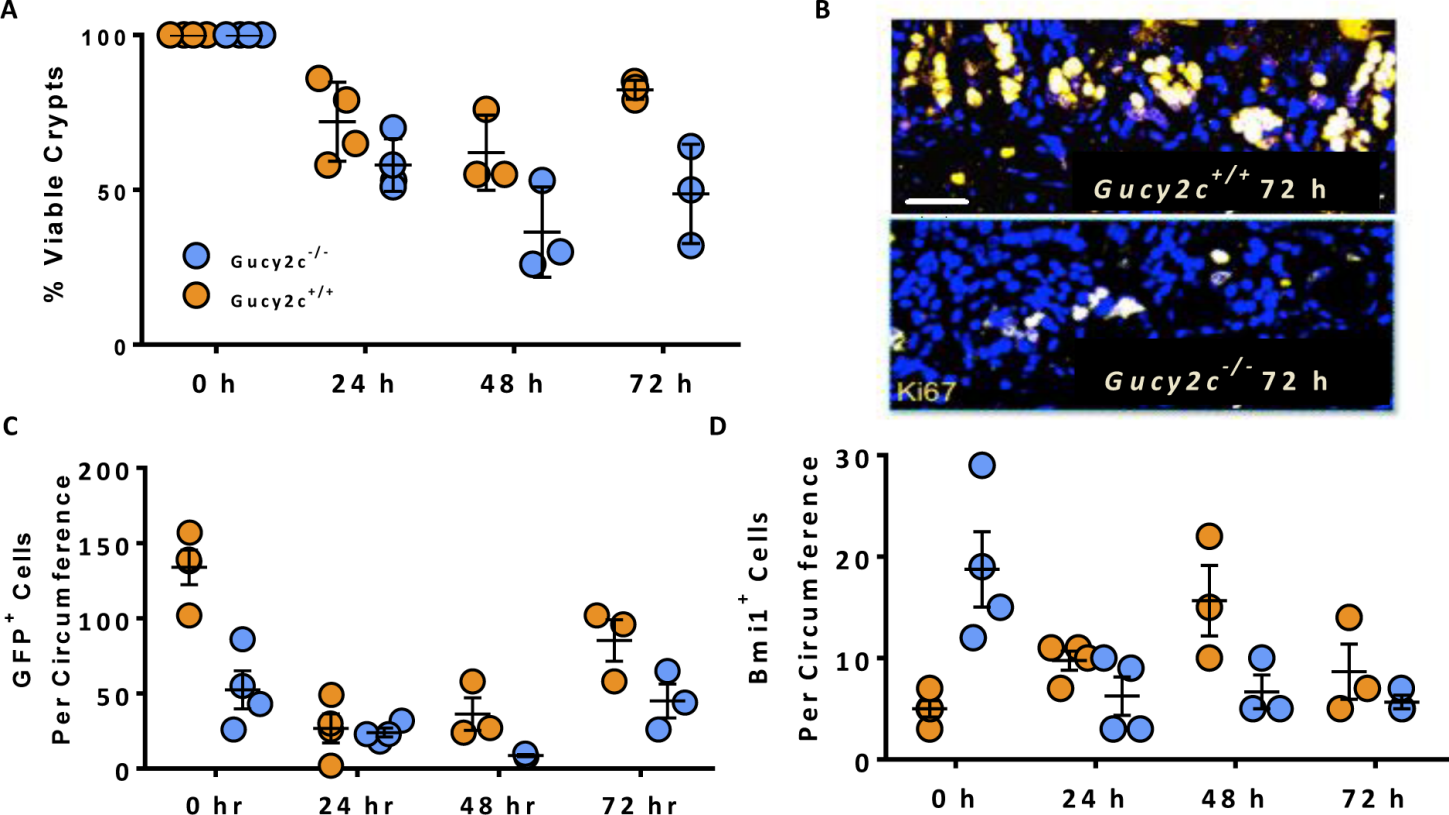
Maintenance of ISCs by GUCY2C contributes to regenerative responses following radiation-induced intestinal injury *Lgr5-EGFP-Cre-Gucy2c*^***+/+***^ and –*Gucy2c*^***–/–***^ mice received 10 Gy of irradiation and the dynamics of (**A**–**B**) viable crypts, (**C**) GFP^+^Lgr5^+^ active stem cells, and (**D**) Bmi1^+^ reserve stem cells were quantified over the subsequent 3 days. Bar in B represents 100 µm.

Intestinal irradiation is an established model to quantify ISC vulnerability and regenerative capacity [36]. Lgr5^+^ cells are exquisitely sensitive to, and depleted by, irradiation while Bmi1^+^ cells are recruited to expand and repopulate the crypt base to support regeneration [6]. A single sub-lethal 10 Gy dose of whole-body radiation produced massive crypt death quantified by the microcolony assay [37] in small intestines of *Gucy2c*^***+/+***^ and *Gucy2c*^***–/–***^ mice (Figure 4A). However, *Gucy2c*^–/–^ mice displayed greater fractional crypt loss compared to *Gucy2c*^***+/+***^ mice 48 hours after irradiation (36% vs 62%, *p* < 0.05), consistent with increased susceptibility to radiation-induced ISC cell death in the absence of GUCY2C signaling (Figure 4A). Further, the absence of GUCY2C signaling was associated with a regenerative lag; *Gucy2c*^***–/–***^ mice recovered only 49% of their crypts compared to 82% in *Gucy2c*^***+/+***^ mice at 72 hours ( *p* < 0.01), consistent with enhanced vulnerability of the crypt in the absence of GUCY2C (Figure 4A–4B). Indeed, the absolute number of Lgr5^+^GFP^+^ cells 48 hours after irradiation was lower (31 vs 9, *p* < 0.05) in *Gucy2c*^***–/–***^, compared to *Gucy2c*^***+/+***^, mice (Figure 4C). In contrast, Bmi1^+^ cells expanded to repopulate the crypt after radiation in *Gucy2c*^***+/+***^ mice, achieving a maximum response at 48 h, while in *Gucy2c*^***–/–***^ mice there was a paradoxical loss of those cells, without recovery, (*p* < 0.01; Figure 4D), paralleling the regenerative lag (Figure 4A). Together, these observations support the hypothesis that GUCY2C signaling, at least in part, protects Lgr5^+^ and Bmi1^+^ stem cells required for regenerative responses to radiation injury.

## DISCUSSION

An emerging paradigm suggests that the crypt harbors populations of multi-potent stem cells which support the unique homeostatic requirements of the continuously regenerating intestinal epithelium. While several intestinal stem cell populations have been suggested, reflecting phenotypic and functional characteristics, there is consensus on two broad categories [38]. Active crypt base stem cells at position 0-4 which are rapidly proliferating and sensitive to insults like radiation are the source of transit amplifying cells which ultimately replace differentiated epithelial cells in routine mucosal maintenance [6, 39]. In contrast, stem cells residing at positions above 4, which are slowly proliferating and relatively resistant to insults, comprise a reserve population that regenerates the intestinal epithelium following injury [40]. While several protein markers have been purported to identify discreet stem cell populations, all are variably expressed by ISCs in crypts [41]. However, Lgr5 and Bmi1 appear to be relatively selective as markers of active and reserve stem cell populations, respectively [38]. This heterogeneity in marker expression likely reflects the plasticity of ISCs. Indeed, rather than discreet stable populations, ISCs likely transition between active and reserve phenotypes to meet the instantaneous needs of normal or injured epithelium [42]. This plasticity creates functional capacity to accommodate wide variations in environmental challenges to the integrity of the mucosa [43]. In turn, this plasticity requires specific mechanisms that maintain the quantity and relative balance of active and reserve stem cells and are only now being discovered.

Here, we reveal that GUCY2C is one key determinant of the quantity and relative balance of active and reserve ISCs. In the absence of GUCY2C, there is a reduction in the quantity of ISCs, reflected in their overall number and in their ability to form enteroids *ex vivo*. Also, there is a shift in the relative balance of these cells with a decrease in active Lgr5^+^ cells and a reciprocal increase in reserve Bmi1^+^ cells. Regulation of the quantity and relative balance of ISCs is associated with the functional co-expression of GUCY2C in stem cells. In that context, reconstitution of cGMP signaling by oral delivery of 8Br-cGMP in *Gucy2c*^***–/–***^ mice restored the quantity and relative balance of active and reserve stem cells. Eliminating GUCY2C is associated with chronic ER stress in crypts, a process associated with loss of stem cells in intestine [34, 44]. ER stress may contribute to ISC loss in *Gucy2c*^***–/–***^ mice since 8Br-cGMP or TUDCA, a chemical chaperone [35], resolved ER stress and restored the quantity and balance of Lgr5^+^ and Bmi1^+^ stem cells. Importantly, silencing GUCY2C increased ISC vulnerability, stem cell loss, and epithelial injury and delayed regeneration in *Gucy2c*^***–/–***^ mice exposed to sub-lethal doses of radiation. These observations highlight a previously unknown role for GUCY2C in maintaining and balancing pools of active and reserve stem cells which, in turn, impacts regenerative epithelial responses to environmental insults.

Mechanisms regulating ISC pools by GUCY2C are likely complex and multi-factorial. Generally, GUCY2C effects are mediated by luminocentric paracrine and autocrine signaling driven by the hormones guanylin and uroguanylin [19]. In ISCs, this regulation may be mediated selectively by guanylin, whose mRNA is expressed in intestinal crypts [45]. The effects of hormone signaling may be cell-autonomous, mediated directly by ISCs, which express GUCY2C in apical membranes making them accessible to luminocentric hormone secretion. Alternatively, these effects may be non-autonomous reflecting the essential role of Paneth cells in maintaining ISCs [2, 8, 23, 36] and the loss of those cells when GUCY2C is silenced [14]. Also, loss of ISCs in the absence of GUCY2C may reflect the associated ER stress, which exits stem cells out of the active Lgr5^+^ pool and into the proliferating progenitor (transit amplifying) pool as part of the canonical differentiation program that renews the intestinal epithelium [34]. Indeed, these observations provide one mechanistic explanation for expansion of the proliferating progenitor cell compartment in intestinal crypts in *Gucy2c*^***–/–***^ mice [13–15, 20, 29]. Further, loss of ISCs in the absence of GUCY2C may reflect an increase in stem cell vulnerability to environmental insults, again likely reflecting the associated chronic ER stress which amplifies stem cell susceptibility to apoptosis [44]. In that regard, GUCY2C signaling enhances resistance of intestinal epithelial cells to chemical, inflammatory and radiation-induced injury [14, 18, 21, 46-48]. Moreover, here we reveal that active Lgr5^+^ cells and reserve Bmi1^+^ cells, which are typically resistant to insults [6], are sensitized to radiation injury in the absence of GUCY2C signaling. Beyond exiting stem cells from the ISC pool and amplifying their vulnerability, the impact of GUCY2C signaling on the plasticity of ISCs and their ability to shift between active and reserve pools remains to be defined. In that context, while there is a reciprocal increase in the reserve Bmi1^+^ cell pool in *Gucy2c*^***–/–***^ mice, these cells fail to fully compensate for the loss of, or restore, active Lgr5^+^ cells in the normal or irradiated epithelium, respectively. These observations suggest that GUCY2C signaling may play a role in the interconversion of Bmi1^+^ and Lgr5^+^ cells that, in part, defines the functional capacity to regenerate in response to environmental insults.

Based on the present observations, it is tempting to speculate that the role of GUCY2C signaling in pathophysiological mechanisms reflects, at least in part, a contribution of dysregulation of the ISC compartment. The GUCY2C signaling axis is universally silenced in colorectal cancer reflecting loss of expression of guanylin in transforming crypts [49-51]. Conversely, eliminating GUCY2C expression promotes intestinal tumorigenesis [14, 20, 52]. The current pathophysiological paradigm of intestinal cancer suggests that initiating transformational events occur in the stem cell compartment [53]. Further, Bmi1 has been identified as an important transcription factor supporting the transformation of cancer stem cells in a variety of tumors [54, 55]. Moreover, GUCY2C is a key component of mechanisms regulating DNA damage repair [14]. These observations suggest a hypothesis in which loss of guanylin silences GUCY2C, shifting ISC pools from active Lgr5^+^ cells to Bmi1^+^ cells which, in the absence of cGMP signaling, may be particularly vulnerable to genotoxic insults amplifying the risk of transformation and cancer. Similarly, inflammatory bowel disease (IBD) is associated with a loss of components of the GUCY2C signaling axis [56]. Conversely, eliminating GUCY2C signaling amplifies tissue injury and mortality in rodent models of IBD [18, 21, 47, 48]. These data suggest a hypothesis in which the loss of GUCY2C signaling in IBD changes the quantity, balance, and quality of stem cells which, in turn, contributes to their vulnerability to injury and attenuates regenerative responses restoring the damaged epithelium. These considerations suggest previously unanticipated pathophysiological paradigms underlying colorectal cancer and IBD which can be explored in future studies.

Beyond pathophysiology, these observations suggest correlative translational opportunities to develop novel therapeutic and preventive approaches that target ISCs. In that context, there are several oral GUCY2C ligands approved, or in development, to treat chronic constipation syndromes [57]. Lumenal expression of GUCY2C by stem cells highlights the feasibility of targeting this receptor using oral replacement strategies to correct paracrine hormone insufficiencies creating dysfunction in the ISC compartment. Indeed, here the FDA-approved oral GUCY2C ligand linaclotide (*Linzess™*) amplified the enteroid-forming capacity, a measure of stem cell quantity and quality, in wild type mice (see Figure 2H). Further, lumenal GUCY2C ligand replacement attenuates intestinal tumorigenesis in mice, and oral GUCY2C ligands are being examined as a novel chemoprevention strategy for colorectal cancer in humans [52, 58, 59]. Additionally, lumenal replacement of GUCY2C ligands ameliorates inflammation in mice, and these agents are in early clinical development in IBD patients [21, 60]. Moreover, the present study reveals that silencing the GUCY2C axis exacerbates the radiation-induced gastrointestinal syndrome (RIGS), the pathophysiology of which has been largely attributed to damage and death of ISCs [61, 62]. The present observations underscore the potential for therapeutic targeting of this signaling axis using oral GUCY2C ligands to defend the crypt to attenuate or prevent RIGS.

In conclusion, we demonstrate that the guanylate cyclase C (GUCY2C) paracrine signaling axis, a key regulator of intestinal epithelial homeostasis, maintains the integrity and balance of active and reserve intestinal stem cells by modulating endoplasmic reticulum stress. These studies reveal a novel role for GUCY2C in supporting intestinal stem cells. Importantly, they underscore the therapeutic potential of oral GUCY2C ligands to prevent or treat diseases reflecting intestinal stem cell dysfunction, including the radiation-induced gastrointestinal syndrome.

## MATERIALS AND METHODS

### Mice and treatments

*Gucyc*^***–/–***^ (*Gucy2c*^***tm1Gar***^ [63]), *Lgr5-EGFP-CreERT2* (B6.129P2-*Lgr5*^***tm1(cre/ERT2)Cle***^/J; Jax, Bar Harbor, ME, #008875) and *Rosa-STOP*^***fl***^*-LacZ* (B6.129S4-*Gt(ROSA)26Sor*^***tm1Sor***^/J; Jax #003474) transgenic mouse lines were interbred to generate offspring with the desired alleles. All mice were co-housed and *Gucy2c*^***+/+***^ (wild type) littermates with the appropriate alleles were used as controls. Tissues were harvested from adult mice (12-16 wk of age). Cre was induced with a single 200 μL dose of tamoxifen (Sigma; Billerica, MA; T5648) in sunflower oil at 10 mg/ml. Tauroursodeoxycholic Acid (TUDCA, Millipore 580549) treatments were administered daily for 3 d at 100 mg/kg/day intraperitoneally. Mice were exposed to a single 10 Gy dose of whole-body γ-irradiation with a PanTak, 310 kVe x-ray machine and tissues were harvested at noted time points after irradiation. In some experiments, mice were gavaged daily for 7 d with 100 μL of 20 mM 8-cpt-cGMP. Each point in a graph (n) represents one mouse unless otherwise noted. All animal protocols were approved by the Thomas Jefferson University Institutional Animal Care and Use committee.

### Immunohistochemistry and immunofluorescence

Intestines were harvested from mice, fixed in formalin, and embedded in paraffin as previously described [20]. Sections (4 μM) were cut then rehydrated in a sequential ethanol-to-water bath and stained with hematoxylin and eosin or antigen-specific primary and secondary antibodies. Primary antibodies for immunofluorescence included: anti-GFP, anti-Bmi1, and anti-GRP78 (Abcam; Cambridge, MA); anti-phospho VASP Ser239 (Sigma; Billerica, MA); and anti-GUCY2C (prepared and validated in-house) [64]. Secondary antibodies were from Life Technologies (Waltham, MA) and specific to the primary hosts. Tyramide signal amplification [65] was used to detect GUCY2C; secondary antibodies conjugated to horseradish peroxidase were from Jackson Immunoresearch Laboratories (cat #115-035-206 and #111-036-046, 1:1000 dilution), and fluorescein-conjugated tyramine was prepared from tyramine HCl (cat #T2879, Sigma) and NHS-fluorescein (cat #46410, Thermo Scientific) as described [66]. For visualization of Rosa-LacZ lineage tracing, tamoxifen-induced recombinant Cre intestines were prepared as described previously [67]. At least 4 intestinal circumference sections were evaluated per mouse.

### Crypt isolation and culture

Crypt isolation for subsequent analyses (enteroid assay, florescence-activated cell sorting (FACS), immunoblot) was performed using a variation of the chelation dissociation method [68]. Briefly, intestines were harvested, villi were gently scraped off for the small intestine, and tissues were minced and incubated in a 10 mM EDTA/Ca-free, Mg-free Hank’s Balanced Salt Solution (HBSS) on ice for a total of 40 min. Throughout this time, solutions were intermittently shaken by hand at the speed of two shakes/second, supernatant was disposed a total of six times, and fresh EDTA/HBSS was added after each disposal. Tissue was incubated undisturbed for 30 min on ice followed by vigorous pipetting with a 10 mL pipet to dissociate the remaining crypts. Crypts were filtered through a 70 µM strainer and pelleted. For enteroid culture, the same number of crypts for each genotype (ranging from 300–1500 crypts/well) were resuspended in a matrigel droplet (BD, 354230), pipetted briefly with a 1000 μL micropipette, plated in 30 μL, and overlaid with 350 μL of Intesticult media (Stem Cell Technologies, Vancouver, Canada; 06005). For FACS, crypts were incubated in 0.25% trypsin (Thermo Scientific, Philadelphia, PA; 15050065) at 37°C until a single cell suspension was obtained (not more than 10 min). Cells were then filtered a second time using a 40 µM strainer and kept in EDTA solution for sorting.

### Fluorescence-activated cell sorting

Cell populations from *Lgr5-EGFP-CreERT2* mice were collected using a Coulter MoFlo Cell Sorter or analyzed using a BD LSRII. Live cells, determined by forward scatter, side scatter, and propidium iodide (PI, Roche), were gated negatively on CD45 (BD Pharmingen, San Jose, CA), then positively gated on CD24^Low^ (BD Pharmingen) [69, 70]. Finally, cells were gated negatively (for differentiated cells) and positively (for Lgr5^+^ cells) gated on endogenous eGFP fluorescence (Supplementary Figure 1).

### Quantitative reverse transcriptase-polymerase chain reaction (RT-qPCR)

RNA from sorted cells was amplified and reverse transcribed *in situ* using total RNA from the CD45^–^/CD24^Low^/EGFP^+^ population. RNA was amplified using MessageBOOSTER cDNA Synthesis Kit for qPCR (Epicentre, Madison, WI) and then subjected to one-step reverse transcription polymerase chain reaction using TaqMan EZ reverse-transcription polymerase chain reaction Core Reagents and appropriate primer/probes for TaqMan GeneExpression Assays in an ABI 7900 (Applied Biosystems, Norwalk, CT).

### Immunoblot

Protein was extracted as described [52], quantitated using BCA assay (Pierce) and subjected to immunoblot analysis using anti-Bmi1 (Abcam; Cambridge, MA), anti-CHOP, anti-calreticulin, anti-phospho-EIF2α, anti-β-tubulin (Cell Signaling, Danvers, MA) and anti-Grp78 (Abcam). Secondary antibodies were from Santa Cruz Biotechnology (Dallas, TX). Molecular weight markers (Cat. # 10748010, 5 µL per run, or Cat. #LC5800, 10 µL per run) for immunoblot analyses were from Invitrogen (Grand Island, NY).

### Transmission electron microscopy

Pieces (3 cm) of intestinal tissue were placed in fixative containing 2.5% glutaraldehyde, 0.1% tannic acid, and 0.1 mol/L phosphate buffer for 5 min three times and stored at 4°C. Tissues were mounted in plastic blocks, processed through 0.1 mol/L phosphate buffer supplied with 2% OsO4 (Osmium), uranyl acetate, then dehydrated through a graded acetone sequence. After being embedded in Spurrs media, blocks were sectioned and visualized using a FEI Tecnai 12 microscope and images will be captured with an AMT digital camera. Representative electron micrographs of each group were taken (kindly performed by Timothy Schneider, Department of Pathology, Thomas Jefferson University). Cells from at least 30 crypts were enumerated per mouse.

### Statistical analyses

All analyses were conducted in a blinded fashion. Two-tailed student’s *t*-tests were used for single comparisons, and two-way analysis of variance (ANOVA) for multiple comparisons, unless otherwise indicated. Cohort sizes were calculated to be sufficient to detect two-tailed statistically significant differences with 95% confidence and 80% power, assuming unequal variances and allowing for unequal sample sizes between groups. *P* < 0.05 was considered significant. Statistical analyses were performed with GraphPad Prism 6 software. Data represent mean ± SEM.

## SUPPLEMENTARY MATERIALS FIGURES



## Abbreviations

CBC: crypt base columnar
cGMP: cyclic GMP
ER: endoplasmic reticulum
GUCY2C: guanylyl cyclase C
ISC: intestinal stem cells
ST: bacterial heat-stable enterotoxin
