# Supplementary material for: GUCY2C maintains intestinal LGR5+ stem cells by opposing ER stress
Source: Oncotarget. 2017 Oct 26;8(61):102923–33. doi: 10.18632/oncotarget.22084 (PMC5732700; doi:10.18632/oncotarget.22084)
Supplement: Supplementary file 1 [file oncotarget-08-102923-s001.pdf]

GUCY2C maintains intestinal LGR5⁺ stem cells by opposing ER stress

SUPPLEMENTARY MATERIALS

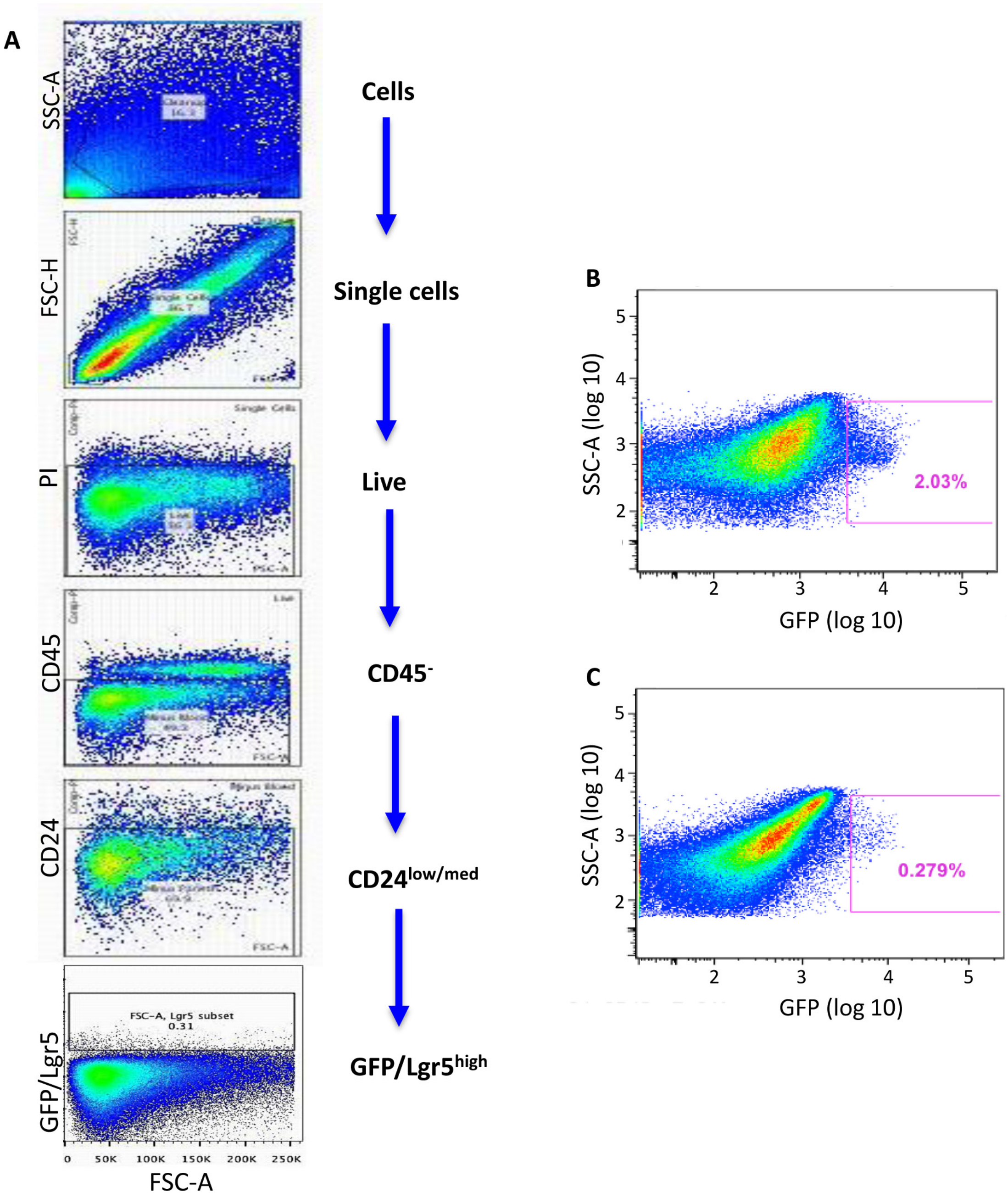

Supplementary Figure 1: Terminal flow cytometry gating strategy for analysis of Lgr5⁺/GFP⁺ cells, with quantification of % GFP^{high} cells. The gating strategy (**A**) was used to identify Lgr5⁺GFP⁺ cells isolated from crypts of (**B**) *Lgr5-EGFP-Cre-Gucy2c*$^{+/+}$ and (**C**) *Lgr5-EGFP-Cre-Gucy2c*$^{-/-}$ mice.

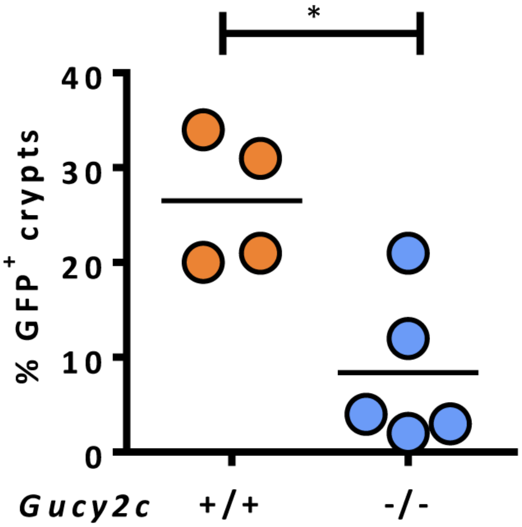

Supplementary Figure 2: Enumeration of intestinal crypts containing Lgr5^{+}GFP^{+} cells (>4 sections/mouse) in *Lgr5-EGFP-Cre-Gucy2c*$^{+/+}$ and *Lgr5-EGFP-Cre-Gucy2c*$^{-/-}$ mice.

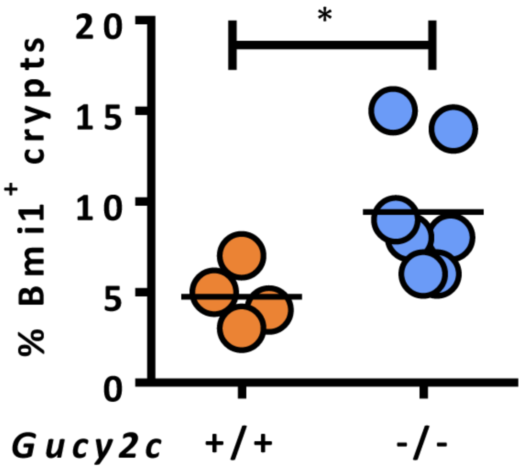

Supplementary Figure 3: Enumeration of intestinal crypts containing Bmi1^{+} cells ($\geq$4 sections/mouse) in *Gucy2c*$^{+/+}$ and *Gucy2c*$^{-/-}$ mice.

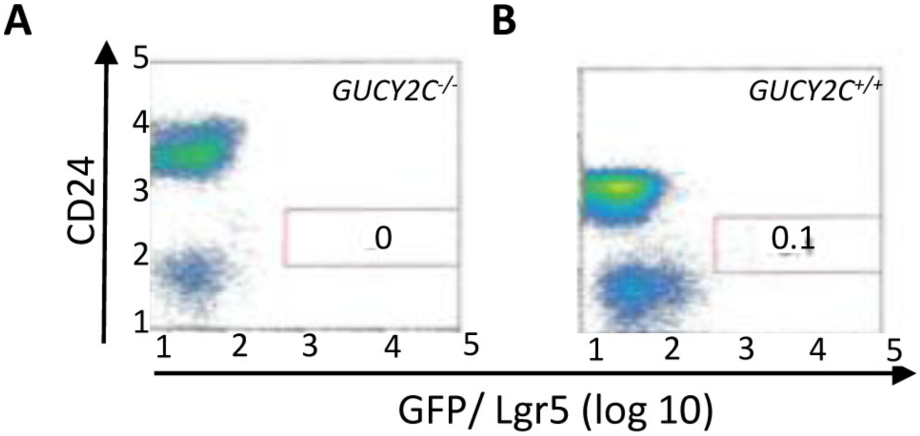

Supplementary Figure 4: Flow sorting strategy of GFP⁻ and GFP⁺ cells from crypts of (**A**) *Lgr5-EGFP-Cre-Gucy2*$^{-/-}$ and (**B**) *Lgr5-EGFP-Cre-Gucy2c*$^{+/+}$ mice.

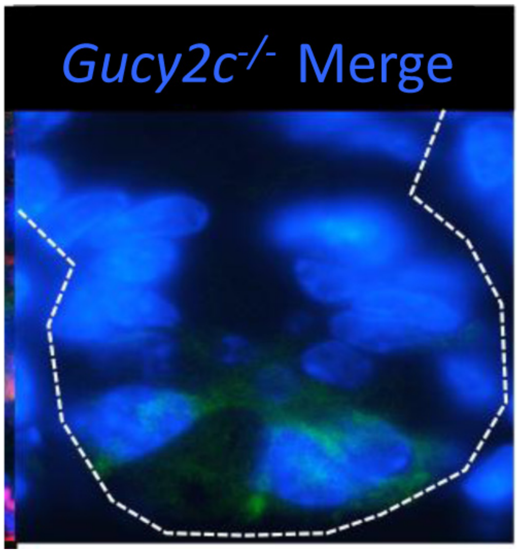

Supplementary Figure 5: Co-staining of GUCY2C and GFP immunofluorescence in GFP⁺ cells in crypts of *Lgr5-EGFP-Cre-Gucy2c*$^{-/-}$ mice. Absence of GUCY2C staining (red) confirms elimination of GUCY2C expression in these mice.
